# Nutritional and Metabolic Requirements for the Infection of HeLa Cells by *Salmonella enterica* Serovar Typhimurium

**DOI:** 10.1371/journal.pone.0096266

**Published:** 2014-05-05

**Authors:** Steven D. Bowden, Amanda C. Hopper-Chidlaw, Christopher J. Rice, Vinoy K. Ramachandran, David J. Kelly, Arthur Thompson

**Affiliations:** 1 Institute of Food Research, Norwich Research Park, Colney, Norwich, United Kingdom; 2 Graduate School of Biological Sciences, Nara Institute of Science and Technology, Ikoma, Nara, Japan; 3 Department of Plant Sciences, University of Oxford, Oxford, United Kingdom; 4 Department of Molecular Biology and Biotechnology, University of Sheffield, Sheffield, United Kingdom; University of Louisville, United States of America

## Abstract

*Salmonella* is the causative agent of a spectrum of human and animal diseases ranging from gastroenteritis to typhoid fever. It is a food - and water - borne pathogen and infects via ingestion followed by invasion of intestinal epithelial cells and phagocytic cells. In this study we employed a mutational approach to define the nutrients and metabolic pathways required by *Salmonella enterica serovar* Typhimurium during infection of a human epithelial cell line (HeLa). We deleted the key glycolytic genes, *pfkA and pfkB* to show that *S*. Typhimurium utilizes glycolysis for replication within HeLa cells; however, glycolysis was not absolutely essential for intracellular replication. Using *S*. Typhimurium strains deleted for genes encoding components of the phosphotransferase system and glucose transport, we show that glucose is a major substrate required for the intracellular replication of *S*. Typhimurium in HeLa cells. We also deleted genes encoding enzymes involved in the utilization of gluconeogenic substrates and the glyoxylate shunt and show that neither of these pathways were required for intracellular replication of *S*. Typhimurium within HeLa cells.

## Introduction

*Salmonella enterica* is one of the most common food-borne bacterial pathogens with disease outcomes in mammals ranging from a self-limited gastroenteritis to typhoid fever. Typhoidal *Salmonella* serovars, such as *Salmonella enterica* serovars Typhi and Paratyphi cause an estimated 20 million cases of salmonellosis and 200,000 human deaths worldwide per annum [1]. *Salmonella* is transmitted via the ingestion of contaminated food and water after which the bacteria penetrate the small intestinal barrier by invading gut epithelial cells causing bloody diarrhoea. In typhoidal salmonellosis, the *Salmonella* pass through the epithelium into the mesenteric lymph nodes where they invade phagocytic cells such as macrophages [2]. Within macrophages, the *Salmonella* bacteria are compartmentalised into a modified intracellular phagosome referred to as the “*Salmonella* containing vacuole” (SCV). The SCV protects the *Salmonella* by preventing lysosomal fusion and exposure to host cell antimicrobial agents [3], [4]. *Salmonella* also resides within an SCV following invasion of epithelial cells [5].

*S*. Typhimurium contains genes related to virulence which enable the *Salmonella* to invade epithelial cells and survive and replicate within macrophages [6]. Most of these virulence factors are encoded by chromosomal genes and many are clustered into Salmonella pathogenicity islands (SPI's). An important SPI cluster is SPI1 which encodes a Type 3 secretion system that is essential for invasion of epithelial cells. The organisation, function and mechanism of action of Salmonella virulence genes, including SPI1 have been extensively studied [7]–[11]. However, relatively little is known regarding the nutritional and metabolic requirements of Salmonella during infection. Recent work has shown that glycolysis and glucose are necessary for the intracellular replication and survival of *S*. Typhimurium in murine macrophages and in BALB/c mice [12]. Other work has shown that gluconeogenesis is not required for infection of BALB/c mice with *S*. Typhimurium strain SR11 and that the tricarboxylic acid (TCA) cycle operates as a full cycle [13], [14]. However it would appear that fatty acid degradation and the glyoxylate shunt are not required to replenish the TCA cycle. The same study also suggested that as yet unidentified sugars are utilised by SR11 for growth during infection of BALB/c mice. In the present study we adopted a mutational approach to examine the nutritional and metabolic pathways required for intracellular replication of *S*. Typhimurium in HeLa epithelial cells. HeLa cells are a well characterised model for *Salmonella* invasion of epithelial cells [15], [16]. We have constructed *Salmonella* strains containing mutations in genes encoding central metabolic pathway enzymes and in sugar transporter systems and show that, in contrast to murine macrophages, glycolysis was only partially required for the intracellular replication of *S*. Typhimurium in HeLa cells, however glucose was a major nutrient source [12]. We also show that gluconeogenesis and the glyoxylate shunt were not necessary for the intracellular replication of *S*. Typhimurium in HeLa cells.

## Materials and Methods

### Bacterial strains, growth conditions and reagents

*S*. Typhimurium strains and plasmids used in this work are listed in Table 1. Strains were maintained in Luria-Bertani (LB) broth or on plates with appropriate antibiotics at the following concentrations; ampicillin (Sigma Aldrich), 100 µg.ml^−1^; chloramphenicol (Cm, Sigma Aldrich), 12.5 µg.ml^−1^; kanamycin (Kn, Sigma Aldrich), 50 µg.ml^−1^; tetracycline (Tet, Sigma Aldrich), 15 µg.ml^−1^. M9 minimal medium with 0.4% w/v glucose was used where indicated. Oligonucleotide primers were purchased from Sigma Genosys or Illumina.

**Table 1:** Strains and plasmids used in this study.

*S*. Typhimurium strains	Relevant genotype	Method of construction	Reference
4/74	Parent strain	N/A	[50]
JH3486	4/74 Δ*pfkA*::Km Δ*pfkB*::Cm	λ Red mutagenesis	[12]
JH3537	4/74 Δ*ptsHI*::Cm	λ Red mutagenesis	[12]
JH3536	4/74 Δ*ptsHIcrr*::Cm	λ Red mutagenesis	[12]
JH3502	4/74 *crr*::Kn	λ Red mutagenesis	[12]
JH3504	4/74 Δ*ptsG*::Cm	λ Red mutagenesis	[12]
JH3494	4/74 *glk*::Kn	λ Red mutagenesis	[12]
JH3541	4/74 Δ*manXYZ*	λ Red mutagenesis	[12]
AT1011	4/74 Δ*ptsG*::Cm, Δ*manXYZ*	λ Red mutagenesis	[12]
AT1012	4/74 Δ*ptsG*::Cm, Δ*glk*::Kn	λ Red mutagenesis	[12]
AT1014	4/74 Δ*ptsG*::Cm, Δ*manXYZ*, Δ*glk*::Kn	λ Red mutagenesis	[12]
JH3469	4/74 Δ*aceA*	λ Red mutagenesis	[14]
JH3487	4/74 Δ*pps*, Δ*pckA*::Kn	λ Red mutagenesis	This study
**Plasmids**			
pKD46	λ Red recombinase expression plasmid	N/A	[17]
pKD3	Cm^R^ resistance cassette-containing plasmid	N/A	[17]
pKD4	Kn^R^ resistance cassette-containing plasmid	N/A	[17]
pCP20	FLP-recombinase expression plasmid	N/A	[17]
pWKS30	Ap^R^ low-copy-number vector, pSC101 origin of replication	N/A	[51]
pWKS30::*pfkA*	Ap^R^ low-copy-number vector, pSC101 origin of replication, expresses *pfkA*	N/A	[12]

### Mutant strain construction

*S*. Typhimurium mutant strains were constructed according to published procedures [17] and as briefly described in [12]. Transductants were screened on green agar plates to obtain lysogen-free colonies [18]. The complete absence of the structural genes was verified by DNA sequencing of the deleted regions of the chromosome. The FLP-recombinase encoded on pCP20 was used to remove the antibiotic resistance markers as described in [17].

### HeLa cell infection assays

Infection assays in human HeLa epithelial cells (obtained from American Type Culture Collection, Rockville, MD) were performed according to [16]. Briefly, HeLa cells were grown in DMEM medium (Sigma, D5546) containing 1 g/L glucose and supplemented with 10% fetal bovine serum (Sigma), 2 mM L-glutamine (Sigma) and 20 mM HEPES buffer (Sigma). Between 1 and 3 x10^5^ HeLa cells were seeded into each well of a 6- or 12-well cell culture plate and infected with *S*. Typhimurium 4/74 and mutant strains at an MOI of 10∶1. Prior to infection the *S*. Typhimurium strains had been grown to an OD_600_ of 1.2 to allow expression of the SPI1 Type 3 secretion system.

To increase the uptake of *Salmonella*, plates were centrifuged at 1000 g for 5 min, and this was defined as time 0 h. After 1 h of infection, extracellular bacteria were killed with 30 µg.ml^−1^ gentamicin. The media was replaced after 1 h with medium containing 5 µg.ml^−1^ gentamicin. Incubations were continued for 2 h and 6 h. To estimate the amount of intracellular bacteria at each time point, cells were lysed using 0.1% SDS, and samples were taken for viable counts [19]. Statistical significances were assessed by using Student's unpaired *t*-test, and a *P* value of <0.05 was considered significant.

## Results

### Glycolysis is partially required for the invasion and intracellular replication of *S*. Typhimurium in HeLa cells

Glycolysis is the sequence of catabolic reactions that converts sugars into pyruvate with the concomitant synthesis of ATP and NADH. It is the foundation of both aerobic and anaerobic respiration and is found in nearly all organisms [20]. The enzyme phosphofructokinase irreversibly converts β-D-fructose 6-phosphate into β-D-fructose1, 6-bisphosphate and is encoded by two genes in most bacteria designated *pfkA* and *pfkB*. [21]. In *Escherichia coli* there are two isozymes of phosphofructokinase (Pfk-1 and Pfk-2). Pfk-1 is a homotetrameric enzyme and the subunits are encoded by *pfkA* [22]. Pfk-2 is a homodimer and the subunits are encoded by *pfkB* [23]. Less than 5% of the Pfk activity in *E. coli* can be attributed to Pfk-2. [24]. The reaction catalysed by phosphofructokinase is a major committing step of the glycolytic pathway and the loss of phosphofructokinase completely blocks glycolysis.

**Figure 1:**
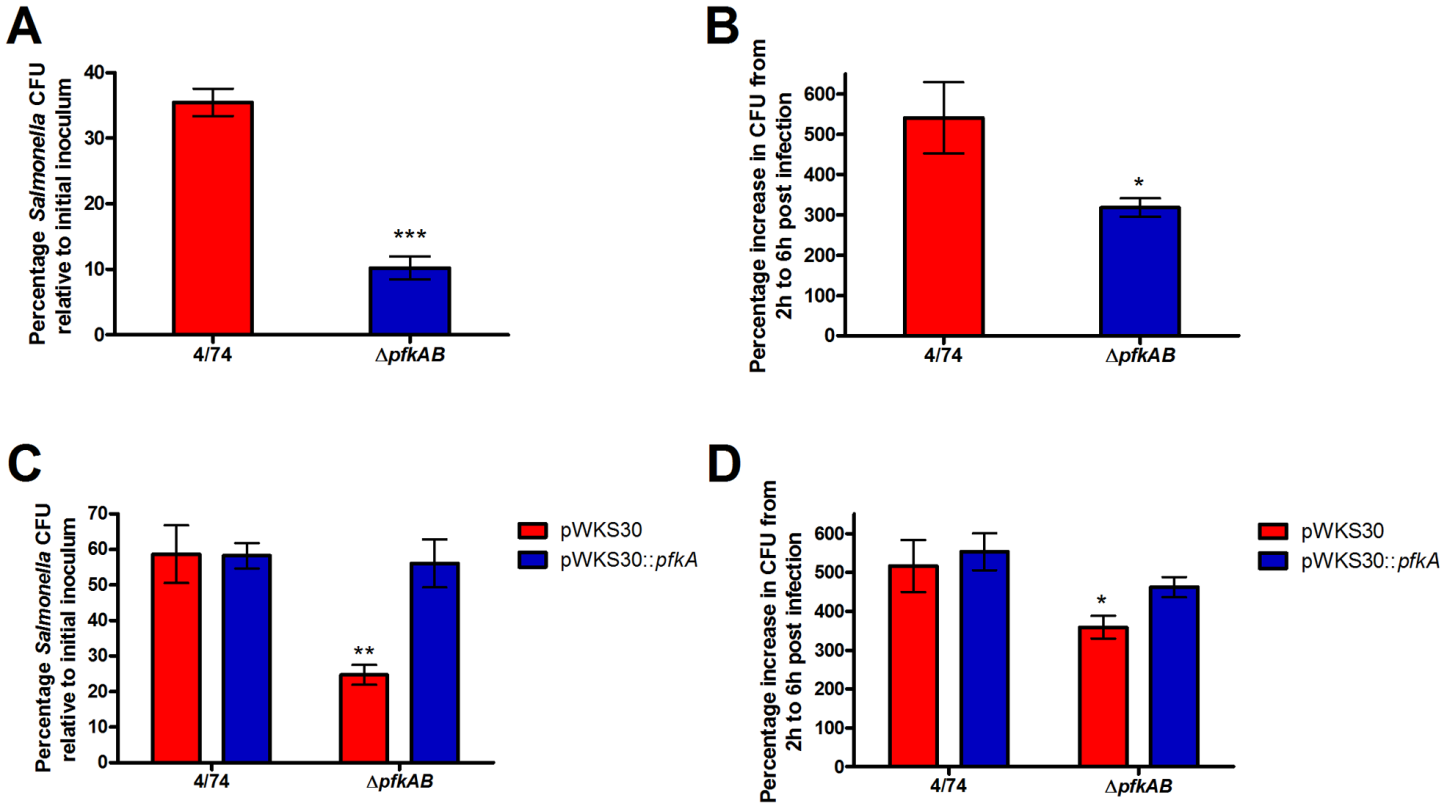
Glycolysis is important but not essential for the invasion and intracellular replication of HeLa cells with *S*. Typhimurium. (A) Invasion assay of *S*. Typhimurium 4/74 parental and Δ*pfkAB* (JH3486) strains in HeLa cells (B). Intracellular replication assays of *S*. Typhimurium 4/74 parental and Δ*pfkAB* (JH3486) strains during infection of HeLa cells. The chart shows the percentage replication of bacteria between 2 h and 6 h. (C) Complementation of invasion of the *S*. Typhimurium Δ*pfkAB* strain in HeLa cells. (D) Complementation of intracellular replication of the *S*. Typhimurium Δ*pfkAB* strain in HeLa cells. Each bar represents the statistical mean from three biological replicates and the error bars represent the standard deviation. (The significant differences between the parental 4/74 strain (A, B), or the 4/74 (pWKS30) strain (C, D) and the mutant strains are shown by asterisks, *p<0.05. **p<0.01, and ***p<0.001).

We tested an *S*. Typhimurium Δ*pfkAB* mutant (JH3486) for its ability to invade and replicate within HeLa cells. We found that the loss of phosphofructokinase reduced the ability of JH3486 to invade HeLa cells (Fig. 1A). Interestingly, the intracellular replication rate of the JH3486 Δ*pfkAB* strain was reduced by only 40% compared to the parental strain (Fig. 1B). This suggests that glycolysis is not absolutely required for the replication of *S*. Typhimurium within HeLa cells which contrasts to that reported previously for *S*. Typhimurium infection of murine macrophages where phosphofructokinase was found to be essential [12]. A Δ*pfkAB* mutant is severely attenuated in its ability to grow within mice; however, this mutant still remains virulent in mice [25]. We next showed that the ability of *S*. Typhimurium JH3486 to invade and replicate within HeLa cells can be fully restored to that of the 4/74 parental strain by complementation with a low copy number plasmid (pWKS30) carrying the *pfkA* gene (Fig. 1CD).

### A transcriptomic analysis of PTS sugar transport gene expression during infection of epithelial cells with *S*. Typhimurium

**Figure 2:**
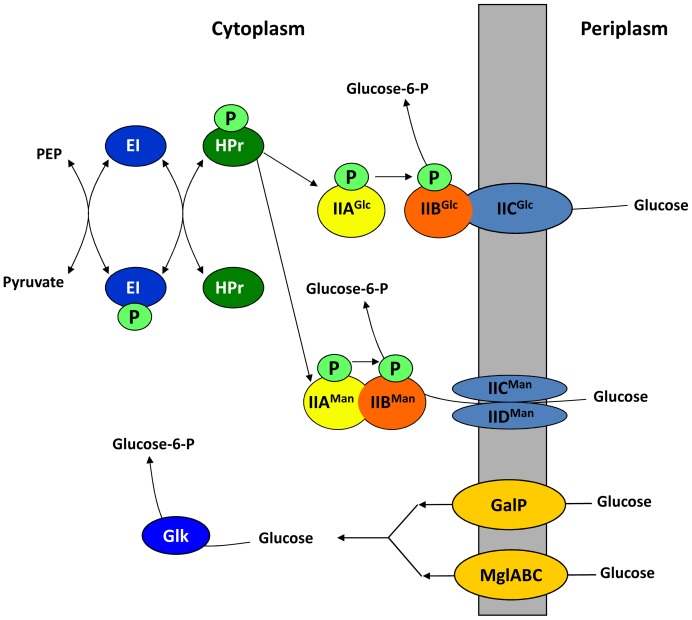
Summary of glucose transport in *Salmonella* [49]. Glucose can be taken up by the EII^Glc^ and/or the EII^Man^ transporters and simultaneously phosphorylated to generate glucose-6-phosphate. The EII^Glc^ PTS transporter is encoded by two genes; *crr* encodes the IIA^Glc^ protein whilst *ptsG* encodes the membrane-bound IIBC^Glc^ protein. The EII^Man^ PTS transporter is encoded by three genes, *manX*, *manY* and *manZ* that encode the IIAB^Man^, and the IIC^Man^ and IID^Man^ components of the transporter system, respectively. In order to transport and phosphorylate glucose, the EII^Glc^ and EII^Man^ transporters require phosphate donated from phosphoenol-pyruvate (PEP) via the EI and HPr phospho-relay proteins that are encoded by *ptsI* and *ptsH*, respectively. In addition, unphosphorylated glucose can be imported by the GalP and/or MglABC transporters then subsequently phosphorylated by glucose kinase (Glk) to produce glucose-6-phosphate.

The observation that loss of phosphofructokinase resulted in a decrease in the intracellular cfu of *S*. Typhimurium within HeLa cells strongly suggested that a sugar catabolised via glycolysis was required for intracellular replication or survival of *S*. Typhimurium. The majority of sugars are transported into *E. coli* and *Salmonella* by the phosphotransferase (PTS) system. The PTS system simultaneously imports and phosphorylates a large number of carbohydrates and seemed a likely candidate to be involved in transporting glycolytic substrates required for growth of *S*. Typhimurium within macrophages and epithelial cells [26]. The PTS system is complex but in summary, phosphoenolpyruvate donates phosphate to enzyme 1 (E1), of the PTS system which in turn passes it on to the histidine protein, HPr. The next step involves a sugar-specific membrane-bound complex, enzyme 2 (EII). Enzyme 2 transports and phosphorylates the incoming sugar and is usually divided into three different domains, EIIA, EIIB, and EIIC [26]. A summary of the PTS system describing glucose transport in *Salmonella* is shown in Fig. 2.

**Figure 3:**
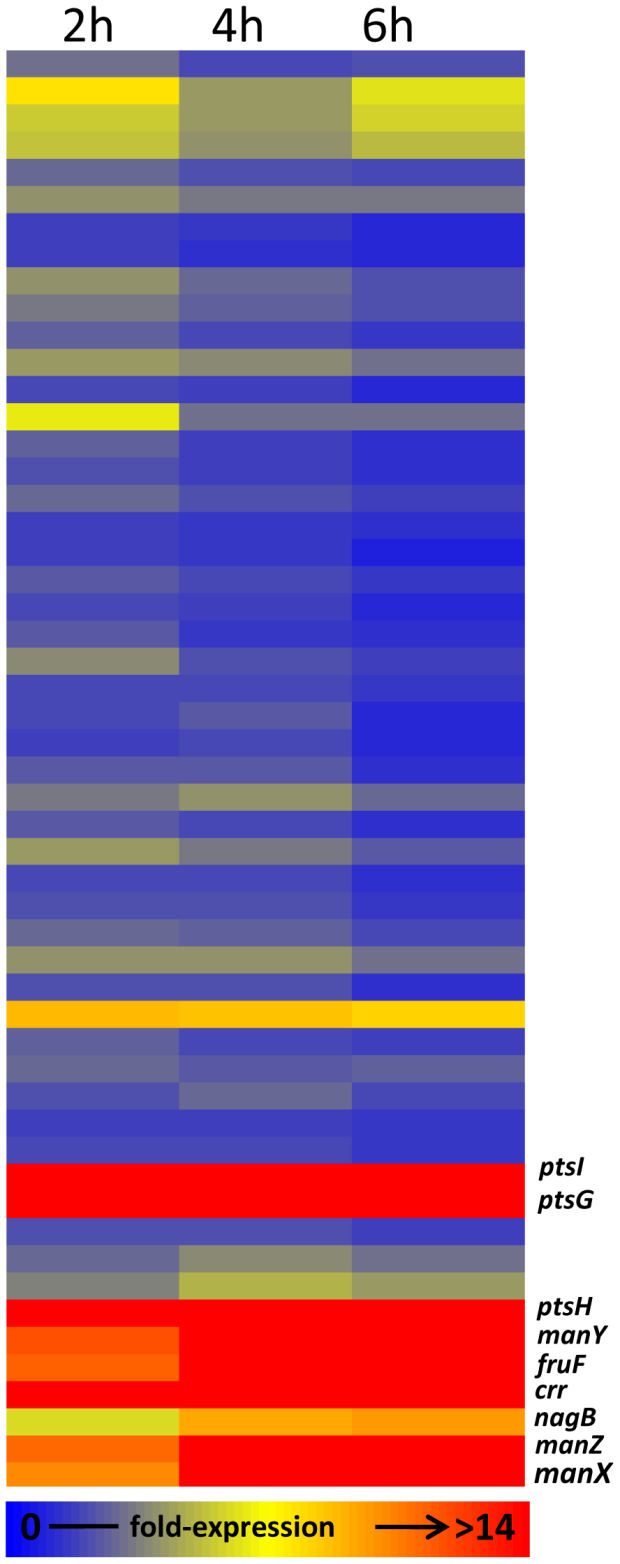
Hierarchical clustering of 53 *S*. Typhimurium PTS genes expressed during infection of HeLa cells. The filtered data was clustered according to similarity of expression level using the standard correlation tool in GeneSpring GX7.3^™^ (Agilent). Each gene is colour-coded according to the level of expression (i.e. signal ratio of cDNA versus genomic DNA). Highly expressed genes are shown in red and weakly expressed genes are dark blue. The clustering map was compiled from microarray data deposited at ArrayExpress (accession number E-MEXP-1368) and described in [16].

We examined the expression profiles of 53 genes encoding components of the PTS system in published transcriptomic data from HeLa cells infected with *S*. Typhimurium [16], [27]. We found 9 PTS genes that formed a distinct highly expressed cluster compared to the rest of the PTS genes during infection of HeLa cells with *S*. Typhimurium (Fig. 3). The genes were *ptsHI, ptsG*, *manXYZ*, *fruF, nagB* and *crr*. The *ptsH* and *ptsI* genes are operonic and encode HPr and E1 of the PTS system respectively. The *crr* gene encodes the enzyme EIIA^Glc^ subunit of the glucose PTS system and its promoter is internal to the *ptsI* gene [28]. The *ptsG* gene encodes EIIBC^Glc^ subunit of the PTS system which is specific for transport of glucose. The *manXYZ* and *fruF* genes encode mannose and fructose specific components of EII respectively. The high expression levels of the *ptsHI, ptsG*, *manXYZ*, *fruF* and *crr* genes compared to the rest of the PTS genes suggested they were likely to play a role in nutrient transport in intracellular *S*. Typhimurium in HeLa cells. Both the *E. coli* and *Salmonella manXYZ* encoded PTS systems are able to transport glucose as well as mannose [29], [30]. We therefore tested whether components of the PTS system encoded by the *ptsHI*, *manXYZ* and *ptsG* genes were required for infection of epithelial cells by *S*. Typhimurium.

### Carbohydrate transport is partially required for invasion and intracellular replication of *S*. Typhimurium in HeLa cells

**Figure 4:**
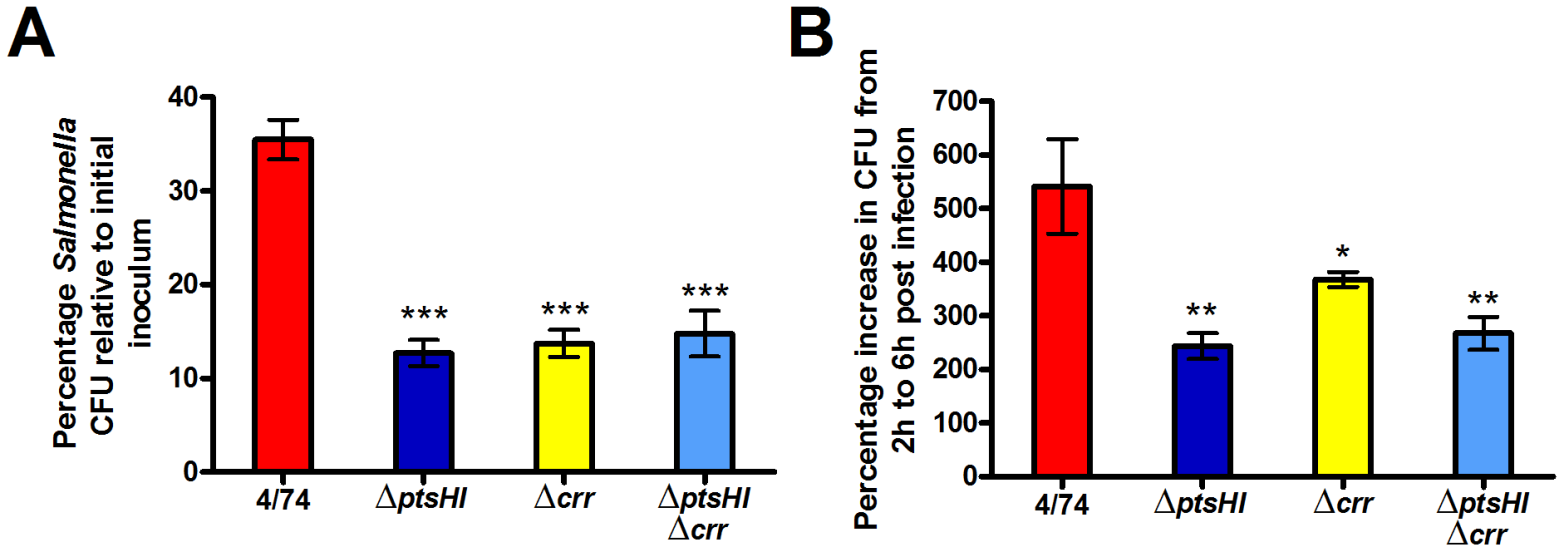
The PTS-system is important but not essential for the invasion and intracellular replication of HeLa cells in *S*. Typhimurium. Invasion (A) and intracellular replication (B) of *S*. Typhimurium 4/74, Δ*ptsHI* (JH3537), Δ*ptsHIcrr* (JH3536) and Δ*crr* (JH3502), strains during infection of HeLa cells. (A) The chart shows the numbers of viable bacteria (expressed as percentages of the initial inoculum) within host cells at 2 h post-infection. (B) The chart shows the percentage replication of bacteria between 2 h and 6 h. Each bar represent the statistical mean from three biological replicates and the error bars represent the standard deviation (The significant differences between the parental 4/74 strain and the mutant strains are shown by asterisks *p<0.05, **p<0.01, and ***p<0.001).

We performed HeLa cell infection assays with *S*. Typhimurium parent strain (4/74), Δ*ptsHI*, Δ*ptsHIΔcrr, and* Δ*crr* deletion mutants of *S*. Typhimurium. We observed that the mutant strains were impaired for invasion of HeLa cells compared to the parent strain (Fig 4A). We also observed that the intracellular cfu of the mutants were significantly reduced compared to the parent strain in HeLa cells (Fig. 4B). The reduced intracellular cfu's of the Δ*ptsHI*, Δ*ptsHI*Δ*crr* and Δ*crr* mutants and the increased expression of the *manXYZ* and *ptsG* genes in HeLa cells (Fig. 3) strongly suggested that glucose was an important substrate for the intracellular replication of *S*. Typhimurium in HeLa cells.

**Figure 5:**
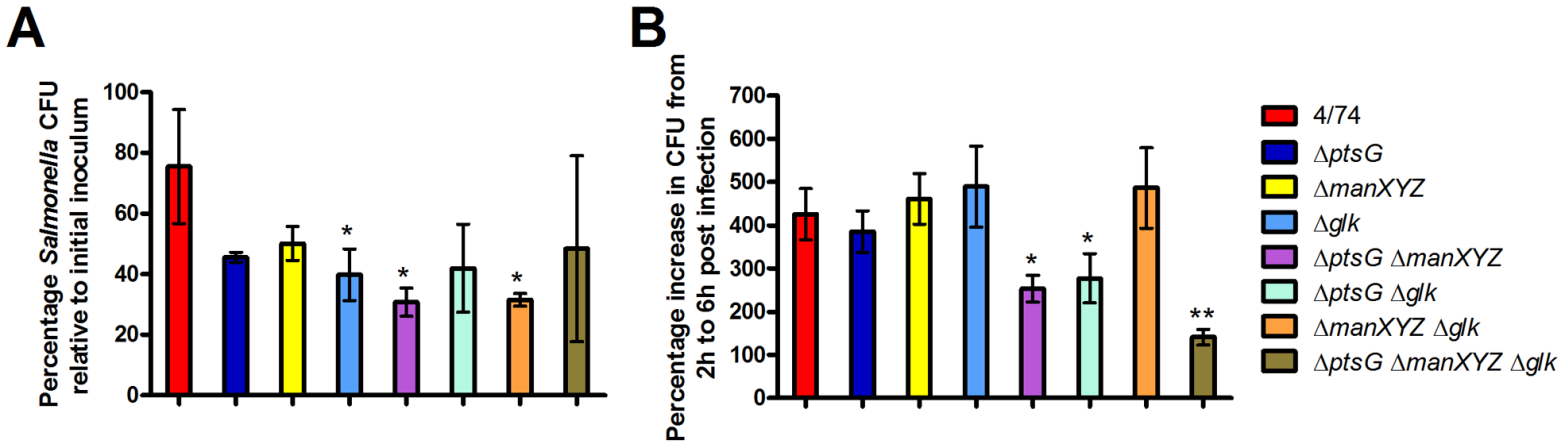
Glucose transport is required for efficient intracellular replication of S.Typhimurium in HeLa cells. Invasion (A) and intracellular replication (B) of *S*. Typhimurium 4/74, Δ*ptsG* (JH3504), Δ*manXYZ* (JH3501), Δ*glk* (JH3494), Δ*ptsG*Δ*manXYZ* (AT1011), Δ*ptsG* Δ*glk* (AT1012), Δ*manXYZ* Δ*glk* (AT1013), and Δ*ptsG*Δ*manXYZ*Δ*glk* (AT1014) strains during infection of HeLa cells. (A) The chart shows the numbers of viable bacteria (expressed as percentages of the initial inoculum) within host cells at 2 h after infection. (B) The chart shows the percentage replication of bacteria between 2 h and 6 h. Each bar indicates the statistical mean for three biological replicates, and the error bars indicate the standard deviations. The significant differences between the parental 4/74 strain and the mutant strains are shown by asterisks *p<0.05, **p<0.01, and ***p<0.001.

*S*. Typhimurium possesses four separate glucose transporter systems capable of importing glucose: the EIIABC^Glc^ and EIIABC^Man^ PTS transporters [29]; GalP [31], [32]; and the methyl galactose transporter, MglABC [33]. The EIIABC^Glc^ and EIIABC^Man^ PTS transporters are encoded by *ptsG*/*crr* and *manXYZ* respectively. The GalP and methylgalactose transporters import unphosphorylated glucose which is subsequently phosphorylated by glucose kinase, encoded by the *glk* gene [34], [35]. An *S*. Typhimurium Δ*ptsG*Δ*manXYZ*Δ*glk* mutant is therefore unable to transport or phosphorylate glucose and we confirmed that it is unable to grow on minimal medium supplemented with glucose as the sole carbon source (data not shown). The Δ*glk* strain showed a slight but significantly reduced (*p*<0.05) invasion phenotype compared to the other two strains and combinations of some of the double mutants (Δ*ptsG*Δ*manXY* and Δ*manXYZ*Δ*glk*) also showed reduced but significant (*p*<0.05) invasion defects (Fig. 5A). Strains carrying individual deletions of either the *ptsG, manXYZ* or *glk* genes showed no significant attenuation within HeLa cells compared to the parent strain (Fig. 5B). The combinations of double mutants carrying the *ptsG* deletion (Δ*ptsG*Δ*manXY* and Δ*ptsG*Δ*glk*) but not the Δ*manXYZ*Δ*glk* mutant were attenuated compared to the parent strain (Fig. 5B), suggesting EIIBC^Glc^ (encoded by *ptsG*) plays a major role in glucose transport. However the triple Δ*ptsG*Δ*manXYZ*Δ*glk* strain showed greater attenuation than any of the combinations of double mutants within HeLa cells (Fig. 5B). The data therefore indicates that glucose is a major carbohydrate source required for intracellular replication of *S*. Typhimurium in HeLa cells and that the glucose-specific PTS system is the principal transport mechanism by which *S*. Typhimurium acquires glucose for intracellular replication in HeLa cells. However, the ability of the Δ*ptsG*Δ*manXYZ*Δ*glk* strain to still replicate within HeLa cells albeit showing significant attenuation compared to the parent strain, may suggest that *S*. Typhimurium is also able to utilize at least one non-PTS substrate to support growth within HeLa cells.

It has recently been shown that *S*. Typhimurium ‘hyper-replicates’ within the cytosolic compartment compared to the SCV in several epithelial cells lines including HeLa cells [36], [37]. One explanation for the continued intracellular replication of the Δ*ptsG*Δ*manXYZ*Δ*glk* strain was that substrates other than glucose may be present (or at higher concentrations) within the cytosol rather than the SCV and that the replication of the Δ*ptsG*Δ*manXYZ*Δ*glk* mutant within HeLa cells represented cytosolic rather than intra-vacuolar replication or *vice versa*. In order to test this possibility we performed infection assays of the parental and the Δ*ptsG*Δ*manXYZ*Δ*glk* strains in the presence and absence of chloroquine (CHQ). Chloroquine specifically inhibits the intra-vacuolar replication of *S*. Typhimurium within host cells (including HeLa cells), and has been used in infection assays to show the hyper-replication of *S*. Typhimurium within the cytosolic compartment [37]. Our data shows that the addition of CHQ decreases the intracellular cfu of the parental strain by 26 +/− 2.5%, and that of the Δ*ptsG*Δ*manXYZ*Δ*glk* strain by 39 +/− 3.3% (Fig. S2). This may suggest that a slightly larger proportion of *S*. Typhimurium Δ*ptsG*Δ*manXYZ*Δ*glk* bacteria were localised to the SCV compared to the parental strain. However, the small difference in the relative distribution of the strains within the host cells suggests that the substrate(s) that permit the replication of the Δ*ptsG*Δ*manXYZ*Δ*glk* strain within HeLa cells are present within both the cytosolic and vacuolar compartments, or may already be present within bacteria prior to invasion.

### The glyoxylate shunt and gluconeogenesis are not essential for intracellular replication of *S*. Typhimurium in HeLa cells

**Figure 6:**
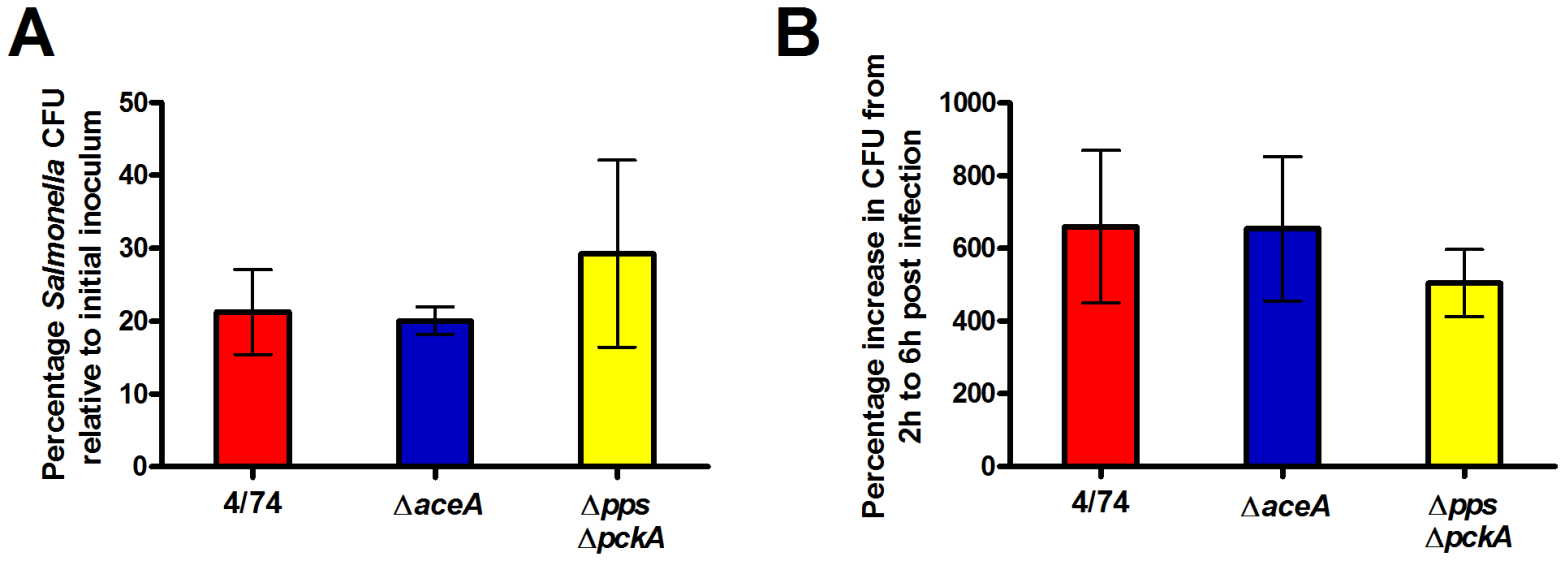
*S*. Typhimurium does not require the glyoxylate shunt or gluconeogenesis for intracellular replication within HeLa cells. Invasion (A) and intracellular replication (B) of *S*. Typhimurium 4/74, Δ*aceA* (JH3385), and Δ*pps*Δ*pckA* (JH3487) strains during infection of HeLa cells. (A) The chart shows the numbers of viable bacteria (expressed as percentages of the initial inoculum) within host cells at 2 h after infection. (B) The chart shows the percentage replication of bacteria between 2 h and 6 h. Each bar represents the statistical mean from two biological replicates (performed in triplicate) and the error bars represent the standard deviation.

We have established that glucose is a major substrate required for intracellular replication of *S*. Typhimurium within HeLa cells; however the intracellular replication of the Δ*ptsG*Δ*manXYZ*Δ*glk* strain (albeit attenuated) could suggest that other substrates might also be able to support replication. We therefore tested the hypotheses that fatty acids metabolized via the glyoxylate shunt were required for the intracellular replication of *S*. Typhimurium in HeLa cells. It has previously been shown that the glyoxylate shunt is required for persistence and replication of the intracellular pathogens, *Mycobacterium tuberculosis*, and the yeast *Candida albicans* [38], [39]. It has also been shown that the glyoxylate shunt is required for the persistence of *S*. Typhimurium in mice [40]. It therefore seemed possible that fatty acids, catabolized via the glyoxylate shunt could be a substrate for intracellular replication of *S*. Typhimurium in HeLa cells. We constructed an *S*. Typhimurium strain containing a deletion of the *aceA* gene which encodes isocitrate lyase. This enzyme catalyses the conversion of isocitrate to succinate and glyoxylate and is essential for the glyoxylate shunt [41], [42]. An *S*. Typhimurium Δ*aceA* mutant was confirmed to be unable to grow on minimal medium with fatty acids as a sole carbon source (data not shown). The deletion of the *aceA* gene had no statistically significant effect on the invasion or the intracellular cfu of *S*. Typhimurium in HeLa cells compared to the parent strain suggesting that the glyoxylate shunt is not required for intracellular replication of *S*. Typhimurium in HeLa cells (Fig. 6).

The intracellular replication of *S*. Typhimurium within HeLa cells could be supported by gluconeogenic substrates (acetate, citrate, malate, succinate, oleate, and pyruvate) [13]. In order to test this possibility we constructed an *S*. Typhimurium strain carrying deletions of the *pps* and *pckA* genes. The *pps* and *pckA* genes encode phosphoenolpyruvate synthase and phosphoenolpyruvate carboxykinase respectively [43], [44]. Pps and PckA catalyse the conversion of pyruvate to phosphoenolpyruvate and oxaloacetate to phosphoenolpyruvate respectively. A Δ*ppsA*Δ*pckA* double mutant is unable to make any gluconeogenic intermediates above pyruvate. The intracellular cfu of the *S*. Typhimurium Δ*pps*Δ*pckA* strain within HeLa cells was slightly reduced compared to the parent strain but this was not significant at *p*>0.05 (Fig. 6B). This result suggests that the utilization of gluconeogenic substrates are not necessary for intracellular replication of *S*. Typhimurium within HeLa cells. One possible explanation for the lack of attenuation of the Δ*pps*Δ*pckA* double mutant and the Δ*aceA* mutant compared to the parental strain was that the presence of sugars enabled growth of the mutant strains within HeLa cells by replenishing TCA cycle intermediates. However, *in vitro* studies in minimal media showed that the addition of galactose (0.01%) did not enhance growth of either the Δ*pps*Δ*pckA* or the Δ*aceA* mutant in the presence of oleate (Fig. S3).

## Discussion

In this study we analysed the central metabolic and nutritional requirements for the intracellular replication and invasion of *S*. Typhimurium in a model human epithelial cell line that has been used extensively to study *Salmonella* infection phenotypes (HeLa). We determined that phosphofructokinase and therefore glycolysis were required, but were not essential, for the intracellular replication and invasion of *S*. Typhimurium in HeLa cells at 6 h post-infection. This is the first demonstration that this major sugar catabolic pathway is required for efficient intracellular replication and invasion of *S*. Typhimurium in an epithelial cell model. These results are in contrast to the discovery that glycolysis was essential for both replication and survival of *S*. Typhimurium in murine macrophages, but not invasion [12]. Interestingly, previous work has identified a set of SPI1 virulence genes whose regulation is effected by the central metabolism of *Salmonella*, suggesting a link between metabolism and invasion efficiency [45].

Glycolysis is the route of entry of a large number of sugars into catabolic pathways. We wished to examine which sugars may be being utilized by *S*. Typhimurium for intracellular replication within HeLa cells. We studied the ability of *S*. Typhimuirum strains carrying mutations in sugar transport genes to replicate within HeLa cells. We found that intracellular replication of an *S*. Typhimurium Δ*ptsHI* and a Δ*ptsHIcrr* strain were reduced by 55% and 51% respectively compared to the parental strain in HeLa cells (Fig. 4B). This suggested that the required carbohydrate was transported partly via the PTS system, and the replication defect of the *S*. Typhimurium Δ*crr* mutant suggested that the required PTS carbohydrate was either glucose, maltose, trehalose, NAG, NAM, arbutin or salicin [28]. Combining the glucose-specific PTS transporter mutants, Δ*ptsG* and Δ*manXYZ* with a glucose kinase mutant (Δ*glk*) revealed that the most important carbohydrate required for intracellular replication of *S*. Typhimurium in HeLa cells was glucose. Since the *S*. Typhimurium Δ*ptsG*Δ*manXYZ*Δ*glk* mutant still replicated within HeLa cells (a 1.4 to 1.9-fold increase in intracellular cfu's between 2 h and 6 h compared to 2.9 to 4.2-fold for the parental strain), it may be that substrates other than glucose can be used for replication of *S*. Typhimurium within HeLa cells.

In order to test the possibility that certain substrates other than glucose could be used to support intracellular replication of *S*. Typhimurium in HeLa cells, we investigated the requirements for fatty acid catabolism and gluconeogenesis. We used an *S*. Typhimurium Δ*aceA* deletion mutant to demonstrate that two carbon compounds do not act as carbon and energy sources for replication of *S*. Typhimurium within HeLa cells. We also showed that gluconeogenic substrates are not necessary for efficient intracellular replication of *S*. Typhimurium in HeLa cells. The observation that neither fatty acids nor gluconeogenic substrates entering below pyruvate were required for intracellular replication, yet Δ*ptsHI* and Δ*pfkAB* mutants were still able to replicate (albeit attenuated compared to the parent strain) may suggest that substrates entering glycolysis at the level of DHAP or GAP (such as glycerol) can sustain replication within HeLa cells in addition to glucose. The potential use of glycerol as a substrate to support *S*. Typhimurium during infection of mice was recently suggested by the observation that *glpFK*, *gldA*, *glpT*, *ugpB* mutants (defective in glycerol utilization) had strong colonization defects [46]. It was also shown from C^13^-isotopologue labeling experiments that *S*. Typhimurium was able to use a C_3_-substrate during infection of Caco-2 cells [47]. However, our data suggests that glycerol utilization does not have a significant impact on intracellular replication of *S*. Typhimurium in HeLa cells since Fig. 4B shows there was no significant differences in intracellular replication between the Δ*ptsHI* and Δ*ptsHI*Δ*crr* strains and *in vitro* growth studies on media containing glycerol as the sole carbon source reveal that the Δ*ptsHI* mutant was highly impaired for growth on glycerol whereas the Δ*ptsHI*Δ*crr* (and Δ*crr*) strain grew on glycerol comparable to the parent strain (Fig. S1). The differences in glycerol utilization between the Δ*ptsHI* and the strains lacking *crr* is due to the inhibition of glycerol uptake by unphosphorylated EIIA^Glc^ in *S*. Typhimurium, as demonstrated by van der Vlag *et al*., 1995 [48].

We also noted that *nagB* showed a similar pattern of expression to the PTS genes in the hierarchical clustering of expression data in *S*. Typhimurium from infected HeLa cells, albeit at a lower expression level (Fig. 2). The *nagB* gene codes for glucosamine-6-phosphate deaminase which deaminates N-acetyl-D-glucosamine (NAG) to ammonia and fructose-6-phosphate. The latter metabolite can enter glycolysis directly suggesting a further possible intracellular substrate. It has also been shown that NagB was one of the most abundant proteins (in excess of 10^5^ molecules per cell) detected in *Salmonella* purified from mouse spleen [46]. However, Fig 4B suggests that NAG does not play a significant role in supporting growth of *S*. Typhimurium in HeLa cells since we only observed slight differences in the intracellular replication of the Δ*crr* strain relative to the and Δ*ptsHI* and Δ*ptsHI*Δ*crr* strains yet the *in vitro* growth phenotypes of the Δ*ptsHI*, Δ*ptsHI*Δ*crr* and Δ*crr* mutant strains in media containing NAG as sole carbon source revealed that only the Δ*crr* strain is able to grow on NAG (Fig. S1). The lack of growth of the Δ*ptsHI* and Δ*ptsHI*Δ*crr* strains on NAG is most likely due to inability to phosphorylate EII^Nag^ and import NAG into the cell [26]. Therefore in conclusion, the attenuation of the Δ*ptsG*Δ*manXYZ*Δ*glk* strain demonstrates that glucose is a major substrate required for intracellular replication of *S*. Typhimurium within HeLa cells, although we cannot rule out that host amino acids may play a role. The observation that glycolysis is not absolutely essential for replication of *S*. Typhimurium within HeLa cells compared to macrophages [12], yet glucose is the major carbohydrate may also suggest glucose catabolic pathways in addition to glycolysis are used by *S*. Typhimurium within HeLa cells. This is a subject of further study.

## Supporting Information

Figure S1 Growth phenotypes of 4/74 parental strain and Δ*ptsHI*, Δ*ptsHI*Δ*crr* and Δ*crr* strains in media supplemented with either glycerol or NAG as sole carbon sources (docx file).
(DOCX)

Figure S2 Chloroquine resistance assay showing cytosolic *vs*. intra-vacuolar replication of *S*. Typhimurium 4/74 parental and Δ*ptsG*Δ*manXYZ*Δ*glk* strains (docx file).
(DOCX)

Figure S3 Growth phenotypes of 4/74 parental strain and Δ*aceA* and Δ*ppsA*Δ*pckA* strains in M9 minimal media supplemented with galactose and/or oleate as sole carbon sources (docx file).
(DOCX)
