# Supplementary material for: Nutritional and Metabolic Requirements for the Infection of HeLa Cells by Salmonella enterica Serovar Typhimurium
Source: PLoS One. 2014 May 5;9(5):e96266. doi: 10.1371/journal.pone.0096266 (PMC4010460; doi:10.1371/journal.pone.0096266)
Supplement: Figure S1 — Growth phenotypes of 4/74 parental strain and ΔptsHI, ΔptsHIΔcrr and Δcrr strains in media supplemented with either glycerol or NAG as sole carbon sources (docx file). (DOCX) [file pone.0096266.s001.docx]

**Figure S1**


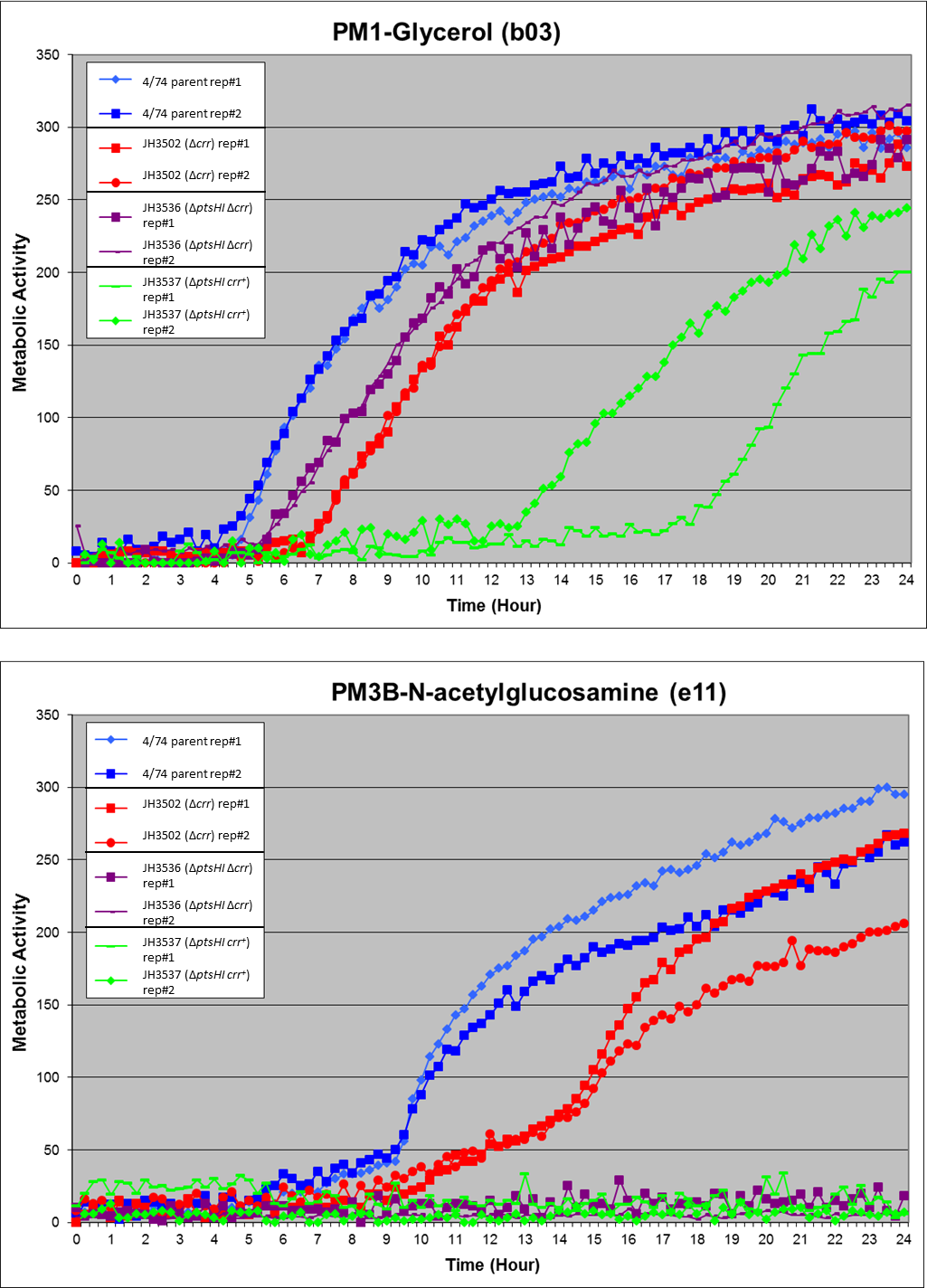


**B**

**A**

**C**


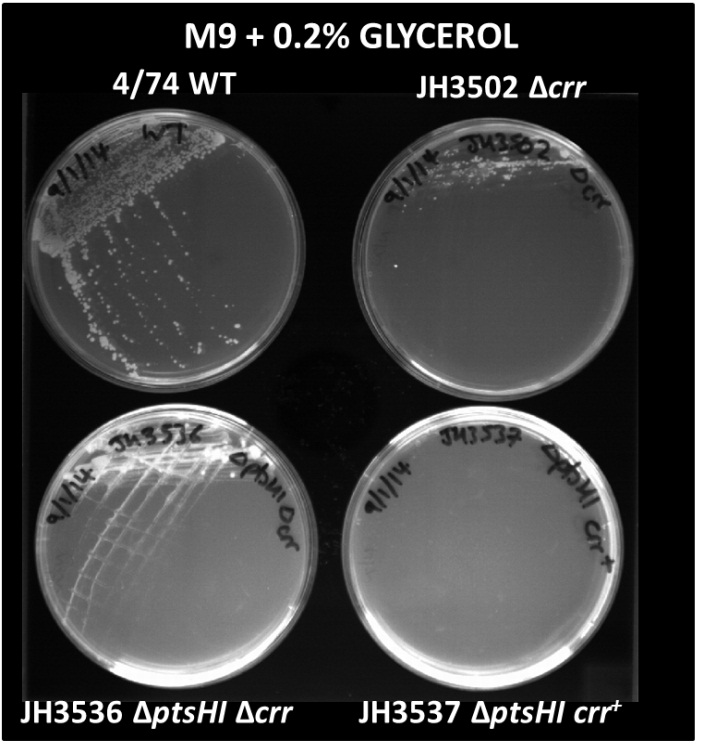
**
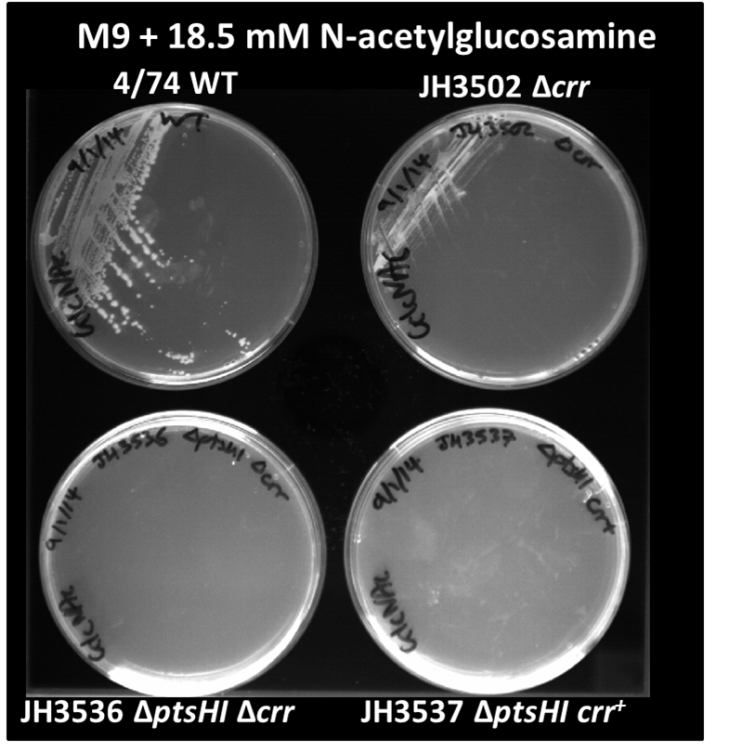
**

**Growth phenotypes of 4/74 parental strain and Δ*ptsHI crr*^+^, Δ*ptsHI*Δ*crr* and Δ*crr* strains in media supplemented with either glycerol or NAG as sole carbon sources.** Results from duplicate phenotype arrays (BiOLOG) showing growth characteristics of 4/74 parental strain (blue) and Δ*ptsHI crr*^+^(green), Δ*ptsHI*Δ*crr* (purple) and Δ*crr* strains (red) in media containing either (A) glycerol or (B) NAG as sole carbon sources. The Δ*ptsHI*Δ*crr* and Δ*crr* show similar growth characteristics to the parent strain (4/74) in media containing glycerol whereas the Δ*ptsHI crr*^+^ strain (green) shows considerably retarded growth. The latter phenotype is likely due to the inhibition of glycerol uptake by unphosphorylated EIIA^Glc^, as described in [48], which can eventually be partially compensated by phosphorylated EII^Nag^. Neither the Δ*ptsHI* *crr*^+^ or Δ*ptsHI*Δ*crr* strains grew in media containing NAG, whereas the Δ*crr* strain (red) did grow on NAG as sole carbon source. This is most likely due to deletion of the *ptsHI* genes resulting in the inability to phosphorylate EII^Nag^ and therefore import NAG into the cell [26]. (C) Confirmation of the growth phenotypes of the 4/74, Δ*ptsHI* *crr*^+^, Δ*ptsHI*Δ*crr* and Δ*crr* strains on M9 minimal media plates containing either glycerol or NAG as sole carbon sources.
