# Supplementary material for: Nutritional and Metabolic Requirements for the Infection of HeLa Cells by Salmonella enterica Serovar Typhimurium
Source: PLoS One. 2014 May 5;9(5):e96266. doi: 10.1371/journal.pone.0096266 (PMC4010460; doi:10.1371/journal.pone.0096266)
Supplement: Figure S2 — Chloroquine resistance assay showing cytosolic vs. intra-vacuolar replication of S. Typhimurium 4/74 parental and ΔptsGΔmanXYZΔglk strains (docx file). (DOCX) [file pone.0096266.s002.docx]

**Figure S2**


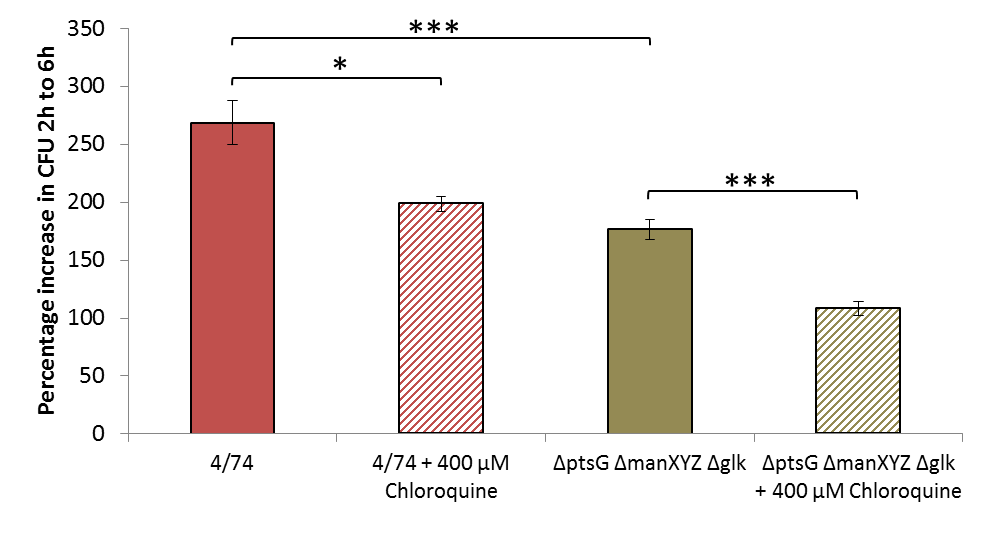


**The proportion of *S*. Typhimurium 4/74 parent and Δ*ptsG*Δ*manXYZ*Δ*glk* strains localised within the cytosol of HeLa cells, as determined using the chloroquine resistance assay.** Intracellular replication of *S*. Typhimurium 4/74 and Δ*ptsG* Δ*manXYZ* Δ*glk* (AT1014) strains during infection of HeLa cells using the chloroquine resistance assay [37]. Infection assays were performed according to the Material & Methods section, with a few alterations. Briefly, HeLa cells were grown in DMEM medium (Sigma) supplemented with 10% fetal bovine serum (Sigma), 2mM L-glutamine (Sigma) and 20mM HEPES buffer (Sigma). Roughly 3 x10^5^ HeLa cells were seeded into each well of a 12-well cell culture plate and infected with either *S*. Typhimurium 4/74 or mutant strain Δ*ptsG* Δ*manXYZ* Δ*glk*, at an MOI of 10:1*.* Prior to infection the *S*. Typhimurium strains had been grown to an OD_600_ of 1.2 to allow expression of the SPI1 Type 3 secretion system. To increase the uptake of *Salmonella*, plates were centrifuged at 1000 g for 5 min, and this was defined as time 0 h. After 1 h of infection, extracellular bacteria were killed with 100 μg.ml^-1^ gentamicin. The media was then replaced with medium containing 10 μg.ml^-1^ gentamicin. Incubations were continued until 2 h and 6 h. At 5 h, media was replaced with medium containing 10 μg.ml^-1^ gentamicin with or without 400 μM chloroquine (Sigma). To estimate the amount of intracellular bacteria at each time point, cells were lysed using 0.1% SDS, and samples were taken for viable counts. The graph shows the percentage increase in bacterial cfu between 2 h and 6 h. Each bar indicates the statistical mean for two biological replicates performed in triplicate and error bars indicate the standard error of the mean. The significant differences between strains and conditions are shown by asterisks, where * *p*< 0.05, ** *p* < 0.01, and *** *p* < 0.001.
