# Supplementary material for: Nutritional and Metabolic Requirements for the Infection of HeLa Cells by Salmonella enterica Serovar Typhimurium
Source: PLoS One. 2014 May 5;9(5):e96266. doi: 10.1371/journal.pone.0096266 (PMC4010460; doi:10.1371/journal.pone.0096266)
Supplement: Figure S3 — Growth phenotypes of 4/74 parental strain and ΔaceA and ΔppsAΔpckA strains in M9 minimal media supplemented with galactose and/or oleate as sole carbon sources (docx file). (DOCX) [file pone.0096266.s003.docx]

**Figure S3**


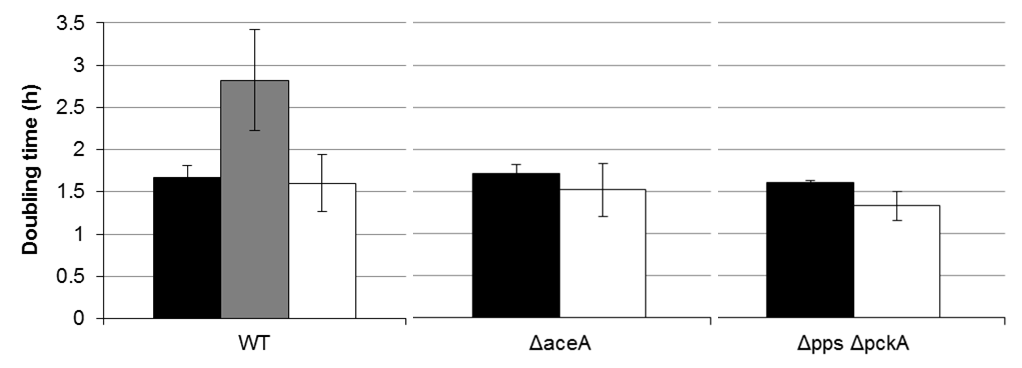


**Doubling times of *S*. Typhimurium 4/74 parental strain, Δ*aceA*, and Δ*pps* Δ*pckA* strains in the presence of a growth limiting concentration of sugar (black bars), fatty acid (grey bar), or a combination of both carbon sources (white bars).** Single colonies of *S*. Typhimurium 4/74, Δ*aceA*, Δ*pps*Δ*pckA* were inoculated into 5 ml LB and grown overnight at 37°C). An overnight culture of each strain was centrifuged (9000×*g*, 5 minutes, 4°C), re-suspended in 5 ml phosphate-buffered saline (pH 7.4) to wash away traces of LB medium, and inoculated via a 100-fold dilution into 25 ml M9 minimal medium, supplemented with either 0.01% (w/v) D-galactose, 5 mM potassium oleate (in 10% Brij-58) or both. Cultures were grown at 37°C with constant aeration over 8 hours. Doubling times were calculated from the exponential portion of the growth curve, corresponding to 1-4 hours post-inoculation. The doubling times of the Δ*aceA* and Δ*pps*Δ*pckA* strains could not be calculated, since neither strain achieved exponential growth using oleate as a sole carbon source.
